# Post-Prandial Amino Acid Changes in Gilthead Sea Bream

**DOI:** 10.3390/ani11071889

**Published:** 2021-06-25

**Authors:** Eleni Mente, Chris G. Carter, Robin S. (Katersky) Barnes, Nikolaos Vlahos, Ioannis Nengas

**Affiliations:** 1 Department of Ichthyology and Aquatic Environment, School of Agricultural Sciences, University of Thessaly, 38446 Fytoko Volos, Greece; 2 School of Biological Sciences, University of Aberdeen, Tillydrone Avenue, Aberdeen AB24 2TZ, UK; 3 Institute for Marine and Antarctic Studies, University of Tasmania, Private Bag 49, Hobart, TAS 7001, Australia; chris.carter@utas.edu.au; 4 University College, University of Tasmania, Locked Bag 1354, Launceston, TAS 7250, Australia; robin.barnes@utas.edu.au; 5 Department of Animal Production, Fisheries and Aquaculture, School of Agricultural Sciences, University of Patras, 30200 Mesolonghi, Greece; vlachosn@upatras.gr; 6 Hellenic Centre for Marine Research, Mavro Lithari, 19013 Anavyssos, Greece; jnegas@hcmr.gr

**Keywords:** amino acids, aquafeeds, fish, single meal, aquaculture, digestion

## Abstract

**Simple Summary:**

Using combinations of plant protein concentrates and EAA supplementation, high levels of replacement (50–75% of fishmeal protein) have been achieved in gilthead sea bream without affecting the growth performance or quality traits. It was confirmed in this study that 16% replacement of marine protein with plant protein meets the amino acid needs of sea bream. The results of the present study suggest the need to further investigate tissue-specific and species-specific responses in the timing and ability to regulate metabolism due to dietary nutrient utilization.

**Abstract:**

Following a meal, a series of physiological changes occurs in fish as they digest, absorb and assimilate ingested nutrients. This study aims to assess post-prandial free amino acid (FAA) activity in gilthead sea bream consuming a partial marine protein (fishmeal) replacement. Sea bream were fed diets where 16 and 27% of the fishmeal protein was replaced by plant protein. The essential amino acid (EAA) composition of the white muscle, liver and gut of sea bream was strongly correlated with the EAA composition of the 16% protein replacement diet compared to the 27% protein replacement diet. The mean FAA concentration in the white muscle and liver changed at 4 to 8 h after a meal and was not different to pre-feeding (0 h) and at 24 h after feeding. It was confirmed in this study that 16% replacement of marine protein with plant protein meets the amino acid needs of sea bream. Overall, the present study contributes towards understanding post-prandial amino acid profiles during uptake, tissue assimilation and immediate metabolic processing of amino acids in sea bream consuming a partial marine protein replacement. This study suggests the need to further investigate the magnitude of the post-prandial tissue-specific amino acid activity in relation to species-specific abilities to regulate metabolism due to dietary nutrient utilization.

## 1. Introduction

Fish growth optimization needs an adequate supply of dietary protein for protein deposition [1,2]. The amount and quality of the dietary protein (i.e., balance of amino acids (AA)) and, in particular, essential amino acids will determine the protein requirement per fish species [3,4]. A low or imbalanced amino acid supply tends to initially stimulate protein synthesis in the liver in order to maintain protein synthesis and growth in the skeletal muscle [2,5]. Dietary amino acid imbalances will lead to increased AA oxidation, increased rates of protein synthesis and turnover and decreased protein retention efficiencies [6]. Regulatory roles of amino acids (e.g., leucine) in the control of protein synthesis [7] and glucose homeostasis [8] have been reported. Protein turnover reflects dietary protein in relation to how closely it matches quantitative and qualitative amino acid requirements [2,9,10]. Protein turnover is clearly of central importance to growth, but only a few studies on fish have investigated protein turnover, protein synthesis and protein degradation in relation to dietary modification and the effect of replacing fishmeal protein [11,12,13,14]. Essential and non-essential amino acids appeared synchronously in the plasma in juvenile rainbow trout (*Oncorhynchus mykiss*) fed a fishmeal diet, while the appearance was less synchronized in fish fed a plant meal diet [15].

Since protein is a costly ingredient in today’s aquafeeds, the determination of dietary amino acid profiles that correctly meet the protein requirement and minimize dietary protein will allow for the optimization of amino acid intake for protein synthesis and their use for energy and other purposes. Thus, this is a vital topic for further research. Considerable knowledge from growth experiments exists about the use of alternative protein sources derived from agriculture production such as corn gluten, extruded pea, lupin, rapeseed and soybean meals as well as consideration of organic and certified ingredients in sea bream aquafeeds [14,16,17,18,19]. However, still little is known about amino acid requirements and amino acid uptake patterns under different dietary plant protein sources and when proteins from different sources are mixed.

Following a meal, a series of physiological changes occurs in animals as they digest, absorb and assimilate ingested nutrients [20]. Changes in free amino acid levels after a meal have been used as a criterion to determine amino acid requirements, based on the hypothesis that the free concentration of an individual amino acid will remain low until its requirements are met [3]. Post-prandial concentrations of free amino acids (FAA) in farmed fish have been intensively studied [2,15,21,22,23,24,25,26,27] and are influenced by nutritional factors including feed intake and diet composition. Nevertheless, there are no studies that have compared the combined effects of dietary protein and time after feeding on sea bream free amino acid levels in vivo.

In a previous experiment, Carter et al. [14] showed that neither protein synthesis rates nor the capacity for protein synthesis were affected by the protein source in sea bream fed diets that contained 63, 55 and 50% fishmeal with 16 and 27% of the fishmeal protein replaced by plant protein in the 55 and 50% fishmeal diets. However, there is a post-prandial nutritional response in the tissues since liver and white muscle rates of protein synthesis were significantly higher at 4–8 h after feeding in comparison to the pre-feeding rates (0 h) and at 24 h and 48 h after feeding. Gomez-Requeni et al. [28] demonstrated that plant proteins in comparison to animal proteins alter FAA pools. Santigosa et al. [29] showed that plant proteins changed the time of the peak of the digestive enzyme activity in sea bream. 

Thus, the present study was conducted to investigate time after feeding combined with the effect of different protein sources on tissue amino acid levels in gilthead sea bream (*Sparus aurata* L.). In addition, this study assesses post-prandial amino acid levels of sea bream fed a marine protein and a plant protein single meal. 

## 2. Materials and Methods

### 2.1. Experiment Setup and Operation 

The experiments were performed at the Institute of Aquaculture, Hellenic Centre for Marine Research, in Athens, Greece. Care and use of animals were approved by the University of Thessaly Departmental Aquatic Animal Ethics Committee (approval number: 003-23/03/2011) and complied with the national guidelines and Directive 2010/63/EU. Sea bream were chosen as they represent a valuable marine carnivorous fish species with a high dietary protein requirement. One hundred and eighty gilthead sea bream (*Sparus aurata*) were stocked in nine cages, three per dietary treatment. Experimental net cages were 2 m^3^ (1 × 2 × 1 m deep) and were suspended in a concrete raceway supplied continuously with filtered seawater. Fish were fed ad libitum by hand 3 times daily at 09:00, 12:00 and 15:00. Three isonitrogenous diets (45% protein) were produced at the Fish Nutrition and Pathology Laboratory, Institute of Aquaculture (Hellenic Centre for Marine Research) in Athens, Greece. The composition of the diets was described earlier by Carter et al. [14]; briefly, three isonitrogenous (45% crude protein) diets were formulated. One fishmeal, high-marine protein diet (ORG) was used containing a 100% sustainable certified Peruvian fishmeal (63%), fish oil (9%) and organic wheat (26.5). One laboratory diet (LAB) containing soybean (10% plant protein) to replace approximately 16% of the marine fishmeal protein, fish oil (9%) and wheat (25.2%) was used. A third diet (COM) containing soybean (16%) and corn gluten protein (7.5%) to replace approximately 27% of the marine fishmeal protein, fish oil (14%) and wheat (11.7%) was used. The organic ingredients follow the organic rules and organic certification, and hence the fishmeal used comes from sustainable certified fisheries. Organic wheat is different from non-organic wheat only in the farming method and not in the crop’s nutritional value.

At the start of the experiment, fish were fasted for 48 h. A group of nine fish (63 ± 3.67 g average weight), one fish per cage, three fish from each dietary treatment, was removed, sacrificed and used as the pre-feeding t = 0 h group for measurements of the free amino acid (FAA) concentrations. The remaining sea bream were fed the three experimental diets. Groups of 9 fish per diet were randomly selected and were removed at 4 h, 8 h and 24 h after feeding. Each fish was individually netted and removed randomly from all the three cages. Wild sea bream of similar size were collected by a local fisherman for tissue amino acid analysis and comparisons. All sea bream were sacrificed by anesthesia overdose (phenoxyethanol), wet weight was measured, liver, gut and white muscle tissues were rapidly dissected out and samples were weighed, frozen in liquid nitrogen and stored in a −80 °C freezer until analysis. 

Liver and white muscle free amino acid levels were measured before feeding and at three times after feeding following a single meal in relation to the effect of partial fishmeal replacement. The amino acid composition of the tissue samples was measured using the method outlined in Lyndon et al. [22] and Mente et al. [9]. The amino acid analysis of the experimental diets is provided in Table 1. The essential amino acid (A/E) ratio [9] of each essential amino acid (EAA) was calculated as the percentage of the total EAA to minimize the effect of different sample pre-treatment and hydrolyzing agents. The amino acid composition of the white muscle, gut and liver of wild sea bream was also measured, and the A/E ratio was calculated for comparisons (Table 1).

### 2.2. Statistical Analysis 

Means with their standard error (SE) are presented. Homogeneity was confirmed using Levene’s test. One-way ANOVA was used to analyze the effect of time after feeding followed by Tukey’s multiple comparison test. Two-way ANOVA was used to analyze the effect of diet and time after feeding on amino acid levels in different fish tissues. If there was no significant interaction between the two factors, the main effects were analyzed. Pearson correlation coefficients between dietary and tissue amino acid composition were calculated. Significance was accepted at 5% or less [30]. All statistical analyses were carried out using SPSS Statistics, version 23.

## 3. Results

In relation to the conventional diet (COM), the laboratory diet (LAB) contained more aspartic acid, glutamic acid, glycine, histidine, arginine, isoleucine, valine and lysine, while the content of serine, proline, tyrosine, leucine and phenylalanine was lower (Table 1). The organic diet (ORG) contained more arginine, lysine and glycine and less leucine, aspartic and glutamic acid than the conventional diet. There were significant differences in amino acid levels of lysine and arginine between the three diets (Table 1) (*p* < 0.05). Concerning the organic diet (ORG), the laboratory diet contained more aspartic acid, glutamic acid, histidine, arginine, isoleucine and valine, while the content of serine, glycine, proline, tyrosine and lysine was lower. 

At the end of the experiment, the free EAA composition of the gut of the fish fed the organic diet was strongly correlated (*r*^2^ = 0.98, *p* < 0.05) with the EAA composition of the fish fed the laboratory diet. The A/E ratios of the white muscle of the wild sea bream showed that the concentrations of arginine, lysine and leucine were the highest (Table 1). The liver of the wild sea bream showed the highest A/E ratios for leucine and isoleucine and similar ratios for arginine, lysine and valine. The EAA composition of white muscle of wild sea bream was more correlated (*r*^2^ = 0.80, *p* < 0.05) with the essential amino acid composition of the laboratory diet than the other two diets. The essential amino acid composition of the liver tissue of wild sea bream was more correlated (*r*^2^ = 0.85, *p* < 0.05) with the essential amino acid composition of the laboratory diet than the other two diets. The EAA composition of the gut of wild sea bream was more correlated (*r*^2^ = 0.91, *p* < 0.05) with the EAA composition of the laboratory diet than the other two diets. The essential amino acid composition of the organic diet was more correlated with the laboratory diet (*r*^2^ = 0.97, *p* < 0.05) than the conventional diet (*r*^2^ = 0.95, *p* < 0.05).

Liver and white muscle free amino acids levels of the pre-feeding (t = 0 h) group were compared with those at 4, 8 and 24 h after feeding to examine whether there were any differences due to diet or time after feeding (Table 2). Diet did not have an effect on post-prandial free amino acid changes (*p*-values, 0.78 for liver, 0.28 for white muscle) in either tissue. However, time after feeding a single meal had a significant effect on the amino acid concentration (*p*-values, 0.001 for liver, 0.0001 for white muscle), for both liver and white muscle tissues. 

The most notable change in the liver was a significant decrease in total AA and total EAA at 4 h after feeding. There were no statistically significant differences (*p* > 0.05) between the mean concentrations of total white muscle FAA before and following feeding (0, 4, 8 and 24 h after a single meal). However, the total essential white muscle FAA were significantly higher at 4 h after a meal (Table 2). The peak amino acid concentrations were not similar between the two tissues but occurred at 4 h after feeding. 

**Figure 1:**
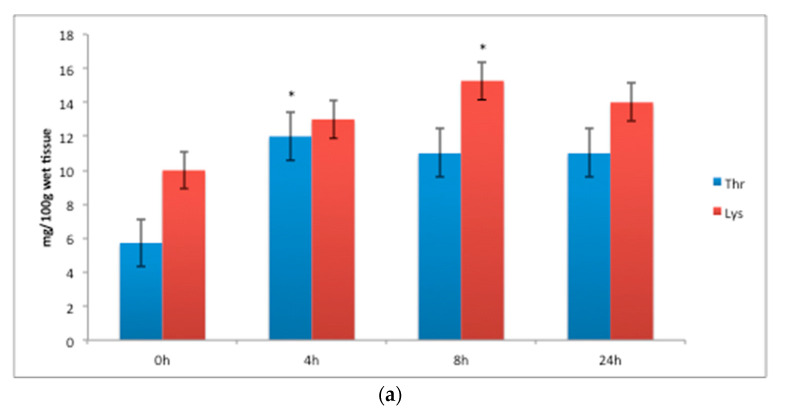

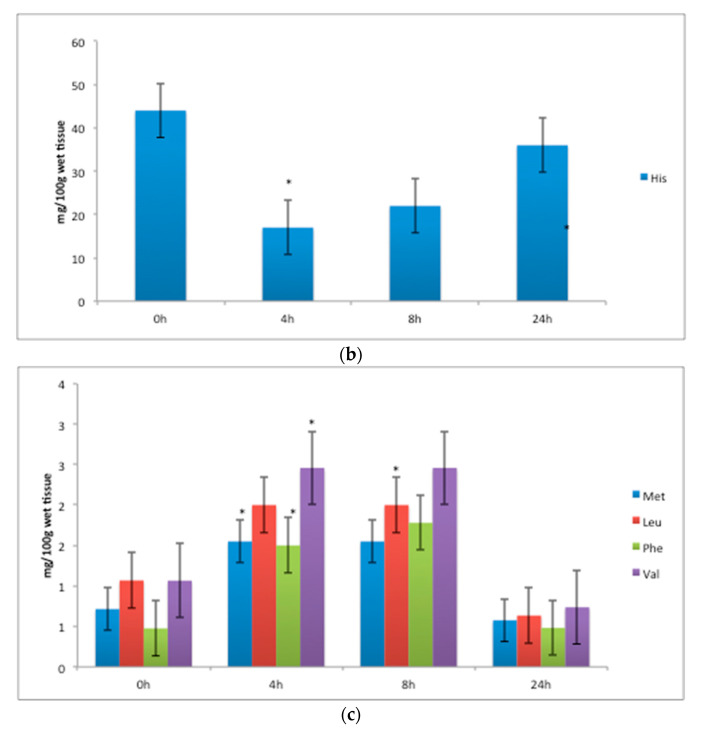
Essential white muscle free amino acid (FAA) concentration (mg/100 g wet tissue): (**a**) threonine and lysine, (**b**) histidine and (**c**) methionine, leucine, phenylalanine and valine before (t = 0 h) and after feeding (4 h, 8 h, 24 h) with the laboratory experimental diet. Data are expressed as mean ± SE, *n* = 9 fish per time point. The asterisk above bars indicates that there are statistically significant differences among the time after feeding (*p* < 0.05).

**Figure 2:**
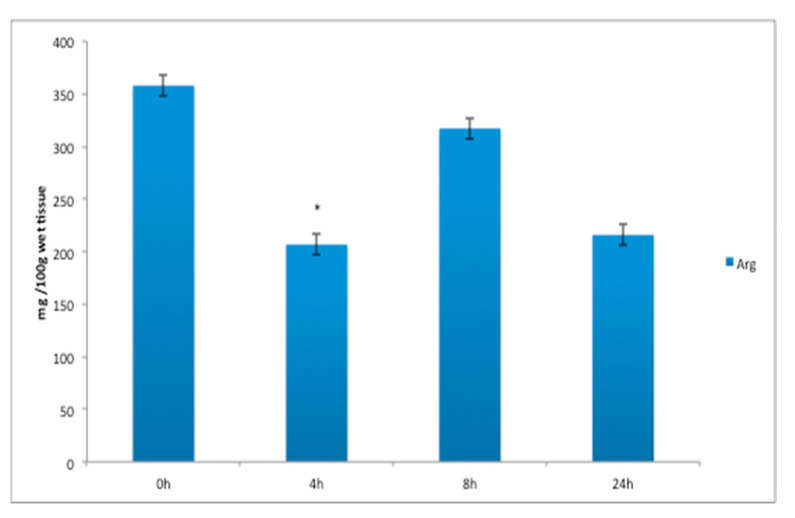

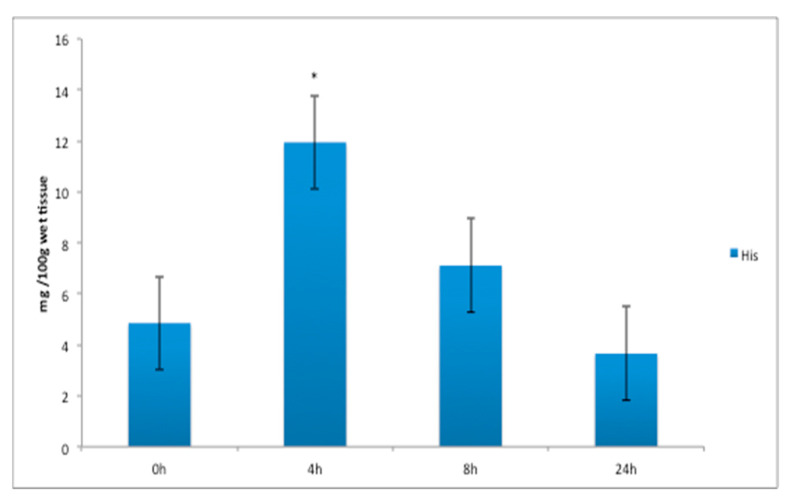
Arginine (Arg) and histidine (His) liver free amino acid (FAA) concentration (mg/100 g wet tissue) before (t = 0 h) and after feeding (4 h, 8 h, 24 h) with the laboratory experimental diet. Data are expressed as mean ± SE, *n* = 9 fish per time point. The asterisk above bars indicates that there are statistically significant differences among the time after feeding (*p* < 0.05).

**Figure 3:**
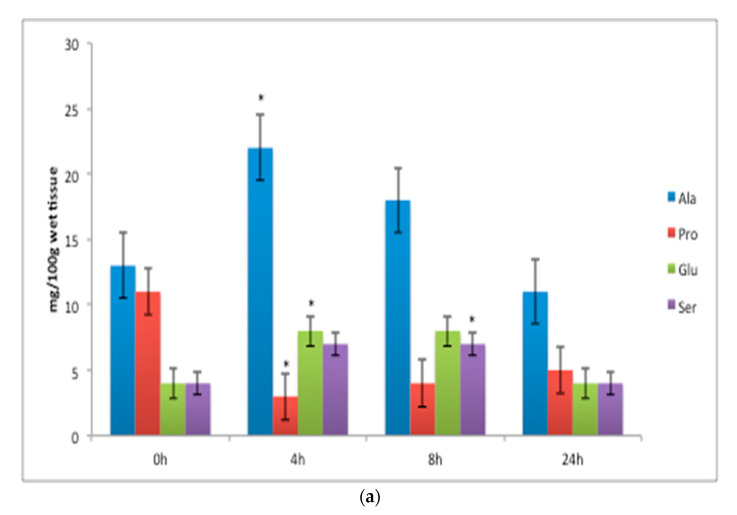

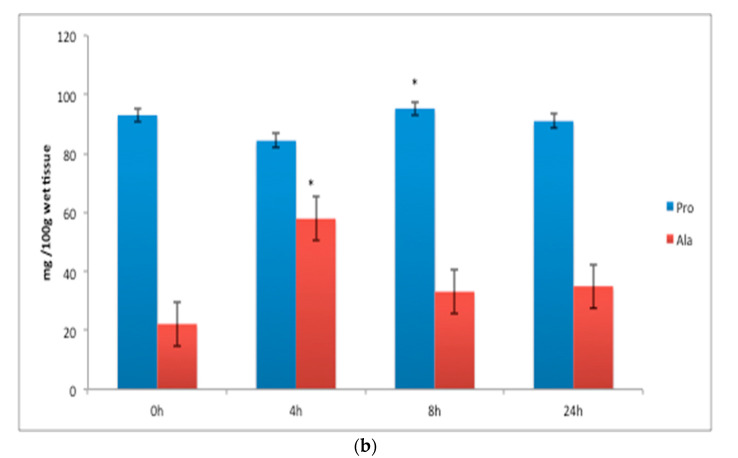
Non-essential free amino acid (FAA) concentration (mg/100 g wet tissue): (**a**) white muscle and (**b**) liver before (t = 0 h) and after feeding (4 h, 8 h, 24 h) with the laboratory experimental diet. Data are expressed as mean ± SE, *n* = 9 fish per time point. The asterisk above bars indicates that there are statistically significant differences among the time after feeding (*p* < 0.05).

The FAA white muscle concentrations of threonine (Figure 1a), increased significantly 4 h after feeding; lysine increased significantly 8 h after feeding (Figure 1a); and histidine (Figure 1b), while the FAA concentrations of valine, methionine and phenylalanine (Figure 1c) increased from 4 to 8 h, and leucine and serine increased significantly at 8 h after a meal (*p* < 0.05) (Figure 1c and Figure 3a). The FAA liver concentrations of histidine (Figure 2) increased significantly 4 h after feeding (*p* < 0.05). Arginine in the liver decreased significantly 4 h after feeding (*p* < 0.05) (Figure 2). The FAA white muscle concentrations of glutamic acid and alanine (Figure 3a) increased significantly 4 h after feeding; proline (Figure 3a) decreased significantly 4 h after feeding (*p* < 0.05). The FAA liver concentrations of alanine (Figure 3b) increased significantly 4 h after feeding (*p* < 0.05). The concentrations of proline in the liver increased significantly at 8 h after a meal (*p* < 0.05) (Figure 3b).

## 4. Discussion

Post-prandial protein synthesis after a single meal in warm- and cold-water fish species increases from 4 to 6 h after a meal, remains elevated and generally returns to the pre-feeding levels after around 24 h [14,20]. Similarly, there are post-prandial changes in free amino acid pools. The pattern of changes in post-prandial protein synthesis is influenced by many factors including the tissue, temperature, diet composition and amino acid intake and specifically by the relative concentrations of FAA in each tissue. Dietary AA are mostly absorbed as FAA or as small peptides [32].

However, tissue concentrations of FAA are tightly regulated [33], and absorbed AA are quickly polymerized into proteins, or irreversibly metabolized. Borey et al. [34] found that the plasma amino acids levels in rainbow trout increased during the post-prandial period, although not significantly in the fish fed a mixed fishmeal/fish oil diet, whereas a significant peak was observed at 6 h after a meal for the plant protein-based diet, and a later response at 12 h was observed in the fish fed the diet rich in fishmeal/fish oil. Figuieiredo–Silva et al. [35] found an increase in plasma FAA 7 h after a meal. In the liver of barramundi, free amino acid levels showed a mild post-prandial elevation, which peaked 2 h after feeding and returned to basal levels within 4 to 8 h [36]. In the present study, in the liver, a significant decrease occurred, whereas in the white muscle, a significant increase occurred, at 4 h after a meal. Time after feeding had a significant effect on amino acids levels after a single meal in sea bream for both the liver and white muscle. There is a number of species-specific responses in the timing and ability to regulate metabolism due to dietary nutrient utilization. Amino acid flux and temporal changes in the free amino acid concentrations in different tissues highlight the dynamic relationship between amino acid intake and amino acid tissue metabolism [22].

A single meal results in an influx of dietary amino acids that is greater than the amount of free amino acids in the whole body, and therefore, due to protein synthesis, tissue free amino acid concentrations change following feeding [33]. The present study has demonstrated that there was some variation in the free pool concentrations of individual amino acids following feeding. However, in the present study, total essential white muscle FAA concentrations increased over a few hours compared to the pre-feeding levels. Tissue free amino acids are known to be affected by dietary protein quality [37,38]. The post-prandial increase 4 h after a meal in the total white muscle EAA pool in fish (Table 2) suggested that most of the muscle amino acids were utilized at a lower rate. Nevertheless, the post-prandial decrease 4 h after a meal in the total liver EAA pool in sea bream showed that there was a high amino acid turnover. Determination of amino acid availabilities following post-prandial changes in tissue free amino acids offers more insight into the fish performance, which was studied earlier in Carter et al. [14]. Carter et al. [23] found an increase in the total free amino acid concentrations in rainbow trout and the largest peaks for the EAA. Following feeding, EAA concentrations in the white muscle in salmonids accounted for 150% of the pre-feeding levels, and the proportion of EAAs did not change significantly [20,39].

In the present study, lysine, valine and leucine concentrations in the white muscle were up-regulated 4–8 h after feeding a single meal. Gilthead sea bream have high dietary arginine, lysine and methionine-cycteine requirements [40]. The A/E ratios of the white muscle of wild sea bream showed that the concentrations of arginine, lysine and leucine were the highest, which correlates with the peak of lysine and leucine found 4 h after a meal. Differences in free amino acids levels being used for protein retention could lead to more amino acids being catabolized due to an imbalance. It seems that intracellular amino acid pools are determined by the movements of amino acids [23]. Thus, due to protein turnover, there is a high demand for free amino acids in the white muscle of sea bream 4 h after a meal.

A good correlation between essential free amino acids in the hepatopancreas or blood plasma and dietary amino acids occurred 4 h after feeding [41]. The liver is the central tissue in the regulation of amino acids, and its FAA pool concentration is influenced by the import and export of amino acids as well as by the use of amino acids within the tissue [42]. Short-term effects of EAA pools in the liver were found in this study. Specific amino acid concentrations in the liver showed a pattern 4 to 8 h after a meal; histidine, proline and alanine showed an increase, while arginine showed a decrease. The decrease in arginine concentrations would suggest that it was rapidly utilized following a meal. The dietary arginine requirements among fish may differ from each other as a result of differences in metabolic and enzymatic efficiency [43]. Arginine plays various physiological roles in animal cells, such as a component of proteins, an oxidative energy substrate, a stimulator of hormone secretion (e.g., growth hormone, insulin, glucagon) and the precursor of polyamine and nitric oxide (NO), which is vital for vasodilation and immune responses [44]. Arginase is ubiquitous in fish tissues, with the highest activity in the liver and kidney [45]. The liver of wild sea bream showed the highest A/E ratios for leucine, isoleucine and similar for arginine, lysine and valine. In sea bream, in the white muscle and liver, Carter et al. [14] found the peak activity in protein synthesis at 4 to 8 h after a meal, and a larger increase in the liver than in the white muscle due to the liver’s primary role in processing ingested nutrients, in the exportation of amino acids and in synthesizing proteins to other tissues. This implies a response to dietary amino acids due to an increase in amino acid metabolism and protein synthesis rates.

The dietary amino acid supply, amino acids’ availability and tissue free EAA levels are related [21], and such interrelation has been used for estimating the amino acid requirements of fish [3,40,46]. Dietary amino acid imbalances will result in unavoidable amino acid losses, and dietary essential amino acid deficiency will cause deamination of the excess amino acids and be used for energy production, lipogenesis or gluconeogenesis, and not for protein synthesis [47]. A balanced dietary amino acid profile has been shown to increase the amino acid retention in Senegalese sole postlarvae [48]. Furthermore, dietary amino acid imbalances affect fish protein growth, especially when the dietary protein content is low or the diet, which was offered to the fish, has a high replacement of marine protein with plant protein [28,49]. In the present study, sea bream were fed three nutritionally balanced, isonitrogenous diets that provided optimum protein and were used to compare the effect of post-prandial tissue amino acids of gilthead sea bream consuming a partial marine protein replacement. We also considered the use of organic certified ingredients. The discussion and the debate on fish feeds certified as organic for organic aquaculture are still open due to the balance that needs to be achieved between the fundamental rules in organic culture and the reality of the supply of feed sources for aquafeeds. In organic fish feed production, following the organic regulation, marine protein and fishmeal are the main protein raw ingredients. The results show that the essential amino acid composition of the high marine protein diet with organic certified ingredients was more correlated with the laboratory diet (r^2^ = 0.97, *p* < 0.05), which contained plant protein to replace 16% of the marine protein, than the conventional diet (r^2^ = 0.95, *p* < 0.05), which contained plant protein to replace 27% of the marine protein.

In this study, following the hypothesis that an efficient diet should have a broadly similar amino acid profile to that of the experimental animal [25], the results show that the laboratory diet seems to have an essential amino acid composition more similar to the white muscle, gut and liver of wild sea bream than the organic and conventional diets. Furthermore, at the end of the experiment, the free EAA composition of the gut of the fish fed the organic diet was strongly correlated (*r*^2^ = 0.98, *p* < 0.05) with the EAA composition of the fish fed the laboratory diet (Table 1). It is rich in histidine, arginine, valine and isoleucine contents and has a good AA balance. In addition, wild gilthead sea bream and organic reared gilthead sea bream reflect the hosted gut prokaryotic communities’ structure [50]. The alternative ingredient quality and the ingredient certification to replace fishmeal protein to be used in organic aquaculture require increased attention. Research continues to evaluate novel formulated alternative ingredients and assess product quality to meet the challenges for the production of organic feeds [51]. Kaushik [46] reported the ideal essential amino acid profile for sea bream, and arginine, lysine and leucine were the three limiting ones. Mente et al. [31] found that the conventional diet had a deficiency in lysine, methionine, valine, histidine and threonine, while leucine and phenylalanine were in excess in comparison to the organic diet. The arginine A/E ratio was similar between the two diets. Takagi et al. [52] showed that soy protein concentrate at an inclusion rate of 50% in the diet was improved by lysine methionine supplementation for juvenile red sea bream. Corn gluten meal can replace 60% of fishmeal protein in diets for gilthead sea bream juveniles, without amino acid supplementation, and was found not to have negative effects on fish performance [53]. There has been much effort devoted to lowering the inclusion rate of marine protein (fishmeal) in aquaculture feeds, and the result of this study suggests that 16% replacement with plant protein does not affect amino acid retention in sea bream.

## 5. Conclusions

Using combinations of plant protein concentrates and EAA supplementation, high levels of replacement (50–75% of fishmeal protein) were achieved in gilthead sea bream without affecting the growth performance or quality traits [54]. It was confirmed in this study that 16% replacement of marine protein with plant-based protein meets the amino acid needs of sea bream. However, higher or even total replacement of dietary fishmeal protein needs further research to evaluate the ability of fish to synthesize each essential amino acid and by developmental stage in species-specific studies. In the present study, lysine, valine and leucine concentrations in the white muscle were higher 4–8 h after feeding a single meal. The post-prandial increase 4 h after a meal in the total white muscle EAA pool in fish suggested that most of the muscle amino acids were utilized at a lower rate. Nevertheless, the post-prandial decrease 4 h after a meal in the total liver EAA pool in fish showed that the amino acid turnover was higher. There is a number of species-specific responses in the timing and ability to regulate metabolism due to dietary nutrient utilization. The results of the present study emphasize the need to further study dietary treatments and the post-prandial time of tissue amino acid activity to improve the nutritional value of diets for fish.

## Figures and Tables

**Table 1:** Amino acid composition (% of total) of the experimental diets and bound essential A/E ratio of white muscle (WM), liver (L) and gut (G) of wild sea bream and gut free essential A/E ratios of sea bream fed the experimental diets.

**Amino Acid (% of Total)**	**Amino Acid Code**		**Diet (COM) ^1^**	**Diet (LAB)**	**Diet (ORG) ^1^**	***p*-Value**	**SE**
Aspartic Acid	Asp		8.10	11.97 *	7.90	0.001	0.150
Glutamic Acid	Glu		12.74	14.79 *	11.84	0.007	0.298
Serine	Ser		5.47	3.52 *	5.02	0.025	0.077
Glycine	Gly		8.63	9.86	10.03 *	0.003	0.106
Histidine	His		1.44	2.11 *	1.63	0.007	0.049
Arginine	Arg		3.90	4.93 *	3.99	0.002	0.110
Threonine	Thr		4.69	4.93	5.20	0.186	0.313
Alanine	Ala		9.62	7.75	10.36	0.104	0.233
Proline	Pro		6.86	4.70	5.81	0.938	0.289
Tyrosine	Tyr		2.59	2.11 *	2.37	0.001	0.034
Valine	Val		6.17	7.04 *	6.55	0.003	0.105
Methionine	Met		2.26	2.82	2.83	0.176	0.235
Isoleucine	Ile		5.06	6.34	5.06	0.185	0.355
Leucine	Leu		10.52	8.45 *	8.87	0.045	0.254
Phenylalanine	Phe		4.89	4.23 *	4.13	0.004	0.068
Lysine	Lys		7.07	7.75	8.36 *	0.001	0.131
**Amino Acid** **(% of Total)**	**Amino** **Acid Code**	**A/E Wild** **WM**	**A/E Wild** **L**	**A/E Wild** **G**	**Free A/E Gut** **Fed the COM Diet**	**Free A/E Gut** **Fed the LAB Diet**	**Free A/E Gut** **Fed the ORG Diet**
Arginine	Arg	10.71	11.80	72.41	78.67	71.43	75.71
Histidine	His	3.28	5.90	4.07	5.03	6.03	4.79
Isoleucine	Ile	11.95	15.61	1.62	0.86	1.22	0.94
Leucine	Leu	14.04	17.70	8.32	1.75	2.68	2.25
Lysine	Lys	15.23	11.80	2.41	1.32	2.15	1.71
Methionine	Met	3.28	5.90	1.22	0.93	1.20	0.61
Phenylalanine	Phe	5.38	7.68	1.63	0.96	1.54	1.27
Threonine	Thr	8.66	5.90	2.78	1.52	2.10	2.25
Tyrosine	Tyr	4.47	5.9	1.41	0.83	1.39	1.10
Valine	Val	11.95	11.8	2.70	1.26	1.66	1.62

The standard error and the *p*-values are taken on the basis of amino acid composition expressed as mg/100 g wet tissue. Data are presented as means of three replicates (*n* = 3). The asterisk indicates that there are statistically significant differences among the diets (analysis of variance, *p* < 0.05; Tukey’s multiple comparison test for means). **^1^** Data from Mente et al. [31]. A/E ratio = {(essential amino acid/total essential amino acids) × 100}. Data are presented as means of three replicates (Mente et al. [9]).

**Table 2:** Free total (AA) and total essential amino acids (EAA) (mg/100 g wet tissue) in liver and white muscle for sea bream before feeding (0 h) and at different times after feeding (4, 8, 24 h) (mean ± SE, *n* = 9 at each time).

Time after Feeding (h)	0	4	8	24
Total AA liver	30.77 ± 0.16 ^a^	24.57 ± 7.17 ^b^	26.11 ± 1.84 ^a^	19.30 ± 3.95 ^a^
Total EAA liver	35.21 ± 1.07 ^a^	31.56 ± 7.91 ^b^	32.51 ± 0.86 ^b^	23.65 ± 2.84 ^a^
Total EAA white muscle	26.56 ± 1.13 ^a^	28.01 ± 2.12 ^b^	25.18 ± 3.50 ^a^	21.76 ± 2.65 ^a^
Total AA white muscle	18.26 ± 5.56 ^a^	19.06 ± 6.52 ^a^	17.04 ± 4.65 ^a^	16.07 ± 6.36 ^a^

There were no statistically significant differences (*p* > 0.05) between the mean concentrations of total AA in white muscle. Mean values in a row followed by a different superscript letter are significantly different (analysis of variance, *p* < 0.05; Tukey’s multiple comparison test for means).

## Data Availability

The data presented in this study are available on request from the corresponding author.
